# Properties of acyl modified poly(glycerol‐adipate) comb‐like polymers and their self‐assembly into nanoparticles

**DOI:** 10.1002/pola.28215

**Published:** 2016-07-08

**Authors:** Vincenzo Taresco, Jiraphong Suksiriworapong, Rhiannon Creasey, Jonathan C. Burley, Giuseppe Mantovani, Cameron Alexander, Kevin Treacher, Jonathan Booth, Martin C. Garnett

**Affiliations:** ^1^ School of Pharmacy, University of Nottingham, University Park Nottingham NG7 2RD United Kingdom; ^2^ Department of Pharmacy, Faculty of Pharmacy Mahidol University Bangkok 10400 Thailand; ^3^ School of Chemical Engineering University of Queensland St Lucia QLD 4067 Australia; ^4^ AstraZeneca, Macclesfield SK10 2NA United Kingdom

**Keywords:** biodegradable polymers, comb‐like polyesters, enzymatic polymerization, grafted polymers, polyester physical–chemical characterizations, poly(glycerol‐adipate)

## Abstract

There is an increasing need to develop bio‐compatible polymers with an increased range of different physicochemical properties. Poly(glycerol‐adipate) (PGA) is a biocompatible, biodegradable amphiphilic polyester routinely produced from divinyl adipate and unprotected glycerol by an enzymatic route, bearing a hydroxyl group that can be further functionalized. Polymers with an average Mn of ∼13 kDa can be synthesized without any post‐polymerization deprotection reactions. Acylated polymers with fatty acid chain length of C_4_, C_8_, and C_18_ (PGAB, PGAO, and PGAS, respectively) at different degrees of substitution were prepared. These modifications yield comb‐like polymers that modulate the amphiphilic characteristics of PGA. This novel class of biocompatible polymers has been characterized through various techniques such as FT‐IR, ^1^H NMR, surface, thermal analysis, and their ability to self‐assemble into colloidal structures was evaluated by using DLS. The highly tunable properties of PGA reported herein demonstrate a biodegradable polymer platform, ideal for engineering solid dispersions, nanoemulsions, or nanoparticles for healthcare applications. © 2016 The Authors. Journal of Polymer Science Part A: Polymer Chemistry Published by Wiley Periodicals, Inc. J. Polym. Sci., Part A: Polym. Chem. **2016**, *54*, 3267–3278

## INTRODUCTION

For many biomedical applications, biodegradability is a key property, especially for injectable formulations. Although all polyesters are endowed with hydrolytically labile linkages in their backbone, only aliphatic polyesters with a short chain in between two ester groups can undergo hydrolysis within a reasonable time frame for biomedical applications.^1^ Amongst aliphatic polyesters the earliest and most widely investigated is the poly(α‐esters) class. The attraction of this class of polymers lies in its immense diversity and synthetic versatility.^2^ Poly(α‐esters) are normally synthesized by poly‐condensation or ring‐opening‐polymerization (ROP) from a variety of monomers. Poly(lactic acid) (PLA)^3^, ^4^ and poly(caprolactone) (PCL)^5^ have been intensively investigated for biomedical applications,^6^ They are approved for pharmaceutical use and have been widely used in the fields of controlled drug release and tissue engineering. Their copolymers can be produced from relatively cheap monomers which can be readily metabolized. Despite these advantages, PLA and PCL materials suffer from some crucial limitations such as slow rate of hydrolysis/degradability due to high hydrophobicity and crystallinity^7^ and their lack of functional group diversity along the polymer backbone. This limits the possibilities for direct control of material properties such as degradation, hydrophilicity, and post‐polymerization modification to enhance drug incorporation and release.^1^ Synthesis of functional lactic and caprolactone monomers is a widely followed route to overcome these drawbacks.^8^, ^9^, ^10^ Unfortunately, this strategy consists of a significant number of synthetic steps of fine organic chemistry and tedious protection/deprotection reactions^11^ requiring labored and time‐consuming purifications that lead to a low yield.^1^, ^9^ There is little pharmaceutical advantage to modifying these particular polymers, as any products will still be classed as new materials and require time‐consuming regulatory approval. More recent bacterial bioprocess routes can be also adopted to produce biodegradable polyesters such as poly(3‐hydroxy alkanoate)s (PHAs).^12^ To improve the chemistry and the amphiphilicity of the materials it is possible either to copolymerize PHAs with other monomers^13^ or engineer bacteria to produce functionalized polymers.^14^ These biosynthetic efforts have yielded materials with higher hydrophobicity which are not favorable in biological applications, while polymers with more hydrophilic moieties are more difficult to produce directly from a biotechnological synthesis and also require post‐polymerization–fermentation steps.^14^ An alternative route to polymers bearing functional groups is through enzymatic synthesis which can avoid the need for protection/deprotection strategies. A further advantage of these biocatalytically synthesized polymers is their potential for enzymatic degradation in biological systems which can be exploited in the same way as other smart polymer modifications. One such enzymatically synthesized polymer is poly(glycerol‐adipate) (PGA) which is typically produced from divinyl adipate and unprotected glycerol yielding polymers with an average molecular weight of 12 kDa (compared with poly(methyl methacrylate) standards). These polymers can be synthesized routinely without any post‐polymerization deprotection reactions in a one‐step synthesis under mild conditions. Materials with a low degree of branching (less than 10% mol/mol) can be produced due to suppression of cross‐linking by steric hindrance at the catalytic site of Novozym 435.^15^ It is possible to change the average molecular weight of PGA *in situ* by changing the polymerization temperature. The resulting PGA is amorphous and in spite of its free hydroxyl groups^15^ is insoluble in water. PGA offers pronounced opportunities as a new biocompatible and biodegradable platform in polymer–drug‐based technologies. The highly tunable branched matrix of PGA, with a pendant hydroxyl group for each repeating unit can readily lead to preparation of comb‐like polymers which have potential applications in biomimetic, pharmaceutical, and biomedical fields. Thanks to the strong repulsion amongst the pendant side chains, grafted‐polymers generally occupy limited space, compared with linear polymers with similar molecular weight and composition, leading to unusual properties.^16^ A more compact structure, with respect to linear architectures, gives rise to higher drug loading and significantly low burst effect, self‐assembling ability and lower critical aggregation concentration (CAC).^16^ PGA and its grafted variants can be used to engineer solid dispersions or micro or nanoparticles (NPs) for healthcare applications. Kallinteri et al.^17^ reported the optimization of the enzymatic synthesis of PGA with various molecular weights together with the incorporation of various amounts of two different acyl substituents, octanoyl chloride, and stearoyl chloride through subsequent modification. These polymers were shown to self‐assemble into NPs with the ability to entrap a model drug such as dexamethasone phosphate with increasing efficiency with Mw and degree of acylation, regardless of the alkyl chain length. In contrast, the polar anticancer drug cytosine arabinoside (CYT‐ARA) showed maximum loading and slowest release from the unsubstituted polymer backbone with the highest molecular weight (12 kDa).^18^ Drug structure and NP formation can strongly affect the degree of interaction and encapsulation in a polymeric matrix. Wahab et al.^19^ showed that long crystalline alkyl chain modifications, at low and high degree of acylation, are not suitable for NP assembly in the presence of Ibuprofen and Ketoprofen. Furthermore, modifying polymers with crystallizable fatty acid chains was shown to partially expel a lipophilic dye, such as Nile Red, from the lipid matrix into the more polar solvent environment.^20^ Weiss et al.^20^ showed that both the nature of the side chain and the degree of substitution affect the structure and aggregation behavior of NPs based on comb‐like polymers. Morphology and size features make an important contribution to the bio‐distribution and transport of NPs *in vivo*.^21^ PGA polymers offer a platform for incorporating a range of pendant substituents which could be of great interest for drug delivery and other biomedical applications. This new family of NPs offers properties vital to lipophilic drug administration, such as the absence of any emulsifier or stabilizer and increased stability due to the cooperative character of the polymer chains.^22^ Both the unmodified PGA and acyl substituted PGA have been shown to have low toxicity on HL‐60 and HepG2 cell lines. Self‐stabilizing NPs of stearyl‐acylated‐PGA with narrow size distributions were shown to have low cytotoxicity.^23^ These results illustrate the possibilities of easily tailoring PGA through simple chemistry to expand its potential in the pharmaceutical and medical fields, for example, in solid dispersions and drug encapsulation. While PGA at low molecular weight (2500 Da) and its stearic modifications have been extensively investigated, only a few physicochemical details for PGA at high molecular weight (over 10,000 Da) and its acyl‐variants can be found in the literature. As highlighted above, high molecular weight polymers would be preferable because of an enhanced drug loading as well as a controlled release.^17^, ^18^, ^24^, ^25^ Therefore, in this study, we modulated the properties of high molecular weight PGA. This was achieved via partial esterification of the pendant hydroxyl functional groups by reaction with the acid chlorides of fatty acids, namely stearoyl (S, C_18_ chain), octanoyl (O, C_8_ chain), and butyryl (B, C_4_ chain) chlorides, producing a plethora of biodegradable/biocompatible comb‐like materials with functionalization degrees ranging from 10% to 90% mol/mol. This set of modifications offers materials with a great range of molecular complexity compared with traditional polymers without functional groups and active moieties. While a number of these modifications have been previously reported, these materials have only been partially characterized. We now report a comprehensive analysis of this novel class of biocompatible polymers through various techniques such as FT‐IR, ^1^H NMR, surface, and thermal analyses. Furthermore, we analyzed the influence of both degree of substitution and side length chain on nanoparticle formation, in the absence of additional surfactants, using dynamic light scattering (DLS).

## EXPERIMENTAL

Novozym 435 lipase ([9001‐62‐1], derived from Candida antarctica and immobilized on an acrylic macroporous resin, solvents, pyridine, and acyl chlorides were purchased from Sigma‐Aldrich (Gillingham, UK), except for divinyl adipate [4074‐90‐2] which was purchased from TCI America. All chemicals were used as received.

### PGA Synthesis

**Scheme 1:**
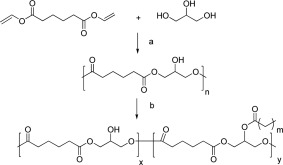
Reagents and conditions: a. Novozym 435, 40 °C, THF, 24 h; b. RC(O)Cl, pyridine, THF reflux, 4 h. Where m can be 2 (C_4_), 6 (C_8_), or 16 (C_18_).

PGA was synthesized by enzymatic (Novozyme 435) reaction (Scheme 1) of divinyladipate (DVA) and glycerol in anhydrous THF for 24 h^17^ as described previously. By keeping both a strictly equimolar stoichiometry ratio of the two monomers, a constant mechanical stirring and by controlling the reaction temperature below 50 °C a reproducible polymer with Mn of 13,000 Da and D of 2.3 can be easily obtained.^15^ For this work all polymers were prepared at 40 °C.

### Acylation of PGA Polymers

General procedure (Scheme 1): synthesis of PGAS20 polymer illustrated. A 100 mL two‐necked round bottom flask, fitted with a condenser, was charged with poly(glycerol‐adipate) (1.00 g, 4.95 mmol) and THF (10 mL). The mixture was heated to reflux temperature to dissolve the polyester, and stearoyl chloride (0.599 g, 1.98 mmol) was added. Pyridine (0.192 mL) was then added dropwise, and a white precipitate of pyridinium chloride formed as the reaction proceeded. The mixture was refluxed for 3–4 h and then cooled down to room temperature, diluted with dichloromethane, added to HCl (2 M, 100 mL) and extracted with dichloromethane (3 × 75 mL). The combined organics were washed with a saturated bicarbonate solution (pH ∼8, 3 × 100 mL) and then with brine (100 mL). The organic layer was dried over magnesium sulfate, filtered, and the solvent removed under reduced pressure to give a hard brown residue, which was kept under vacuum at 50 °C for at least 2 days. C_4_‐ and C_8_‐modified PGAs were isolated as viscous oily residues. To ensure a high degree of purification all the materials was redissolved in THF and precipitated in aqueous solution of NaOH 1 M. All acylated polymers were synthesized from PGA prepared at 40 °C.

### Nanoparticle Formation

The PGA and PGA derivative NPs were prepared by a solvent diffusion evaporation method using acetone as a solvent. The polymeric solution of the polymer (10 mg) in acetone (2 mL) was prepared and added drop‐by‐drop into deionized water (5 mL). The mixture was stirred at room temperature overnight to evaporate acetone. The colloids were collected and further analyzed for their particle size and polydispersity index by Zetasizer Nano ZS (Malvern Instruments Ltd, Malvern, UK). The statistical analysis was performed using IBM® SPSS^®^ Statistics version 21 software.

### Polymer Characterization

#### Instruments

##### FT‐IR Spectroscopy

FT‐IR spectra were recorded with an Attenuated Total Reflection (ATR‐IR) spectrophotometer (Agilent Technologies, Stockport UK, Cary 630 FTIR) equipped with a diamond single reflection unit. Spectra were acquired with a resolution of 4 cm^−1^, in the range 4000–650 cm^−1^ by co‐adding 32 interferograms.

##### ^1^H NMR Spectroscopy

^1^H NMR spectra were recorded on a Bruker 400 MHz spectrometer using acetone‐*d*_6_ as the solvent. Chemical shifts are expressed in parts per million (*δ*). (PGA (400 MHz, acetone‐*d*_6_; *δ*, ppm): 5.31 (m, 1H), 5.10 (m, 1H), 4.90–3.50 (m, 6H), 2.39 (m, 4H), 2.07 (acetone‐*d*_6_), 1.66 (m, 4H). PGAS27 (400 MHz, acetone‐*d*_6_; *δ*, ppm): 5.30 (m, 1H), 5.10 (m, 1H), 4.50–3.50 (m, 6H), 2.39 (m, 6H), 2.07 (acetone‐*d*_6_), 1.66 (m, 6H), 1.31 (m, 28H), 0.9 (m, 3H). PGAO65 (400 MHz, acetone‐*d*_6_; *δ*, ppm): 5.30 (m, 1H), 5.10 (m, 1H), 4.50–3.50 (m, 6H), 2.39 (m, 6H), 2.07 (acetone‐*d*_6_), 1.66 (m, 6H), 1.32 (m, 8H), 0.92 (m, 3H). PGAB45 (400 MHz, acetone‐*d*_6_; *δ*, ppm): 5.30 (m, 1H), 5.10 (m, 1H), 4.50–3.50 (m, 6H), 2.39 (m, 6H), 2.07 (acetone‐*d*_6_), 1.66 (m, 6H), 0.94 (m, 3H).

##### Water Contact Angle.

Water contact angle (WCA) values were measured at 25 °C using a KSV Cam 200 (KSV Instruments Ltd, Helsinki, Finland) equipped with dedicated software (CAM200). Samples were prepared by coating microscopic glass slides with polymer thin films by solvent evaporation of 3 mg/mL polymer solutions in acetone. Four measurements were recorded for each polymer.

##### Differential Scanning Calorimetry.

Polymer thermal properties were investigated by DSC (Q2000, TA Instruments, Leatherhead, UK) at a heating rate of 10 °C/min. Thermal Analysis Software (Version 4.5.05A) was used to analyze the resulting data. Crimped hermetic aluminum pans (TA Instruments, Brussels, Belgium) were utilized for the analysis of the samples under nitrogen flow at rate of 50 mL/min. Glass transitions were analyzed performing four heating/cooling cycles from −90 to 120 °C.

##### Hot Stage Microscopy (HSM).

PGAS was also subjected to heating/cooling cycles in a Hot‐Stage‐Advanced Polarising Microscope (Prior LuxPOLTM with 12V and 30W halogen lamp).

#### AFM Measurements

AFM measurements were carried out by using a Bruker Icon FastScan SPM in PeakForce Quantitative Nanomechanical Mapping (QNM) mode using NFESPA probes (nominal *f* = 75 kHz, *k* = 3 N/m). Probe stiffness and sensitivity was calibrated using a clean glass coverslip, and tip shape was estimated by measuring a standard PS/LDPE sample. Samples were prepared by dissolving polymers in acetone followed by dropping the resulting solutions (5 μL) onto clean glass coverslips.

### Nanoparticle Characterization

Particle size analyses were performed by Dynamic Light Scattering employing a Zetasizer Nano spectrometer (Malvern Instruments Ltd) equipped with a 633 nm laser at a fixed angle of 173°. The results are reported as the z‐average. All experiments were performed in triplicate on the same sample.

## RESULTS AND DISCUSSION

To evaluate the effects of the pendant side chains on the PGA backbone, all its acyl‐modifications underwent a wide range of analyses. Thus, it will be possible to have a better insight of the features and behavior of these materials, their tendency to self‐assemble in water and to establish their applicability in the pharmaceutical field.

### ^1^H NMR Characterization

**Figure 1:**
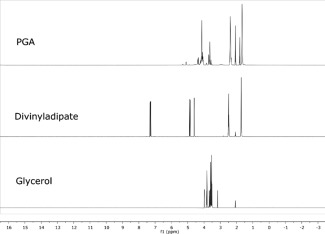
Stacked ^1^H NMR spectra of PGA homopolymer compared with free DVA and free glycerol.

^1^H NMR analysis was used to quantitatively assess the monomer conversion, the molar ratio of the monomers in PGA backbone and the acyl conversion of the pendant alcoholic moiety in the repeat unit. The analysis of the PGA spectrum shows the absence of any DVA vinyl proton signals at 7.29, 4.87, and 4.59 ppm and the appearance of the PGA pattern of methylene and methine signals (Fig. 1), indicating a quantitative conversion of the monomers. The PGA ^1^H NMR spectrum can be divided into two sub areas, in particular, –C*H*_2_ adipic protons were present in the spectral range between 1.6 and 2.5 ppm whilst all protons related to glyceride repeating units were found between 3.5 and 4.5 ppm. Peaks found at 5.10 and 5.30 ppm revealed the presence of 1,2‐disubstituted and 1,2,3‐trisubstituted glycerides, respectively. As previously reported^15^ the presence of the peak at 5.30 ppm (c″) is related to the branching formation due to lack of regioselectivity of the enzyme during the reaction. The PGA backbone used in this work showed a branching amount lower than 9% mol/mol (calculated by ^1^HN MR).

**Figure 2:**
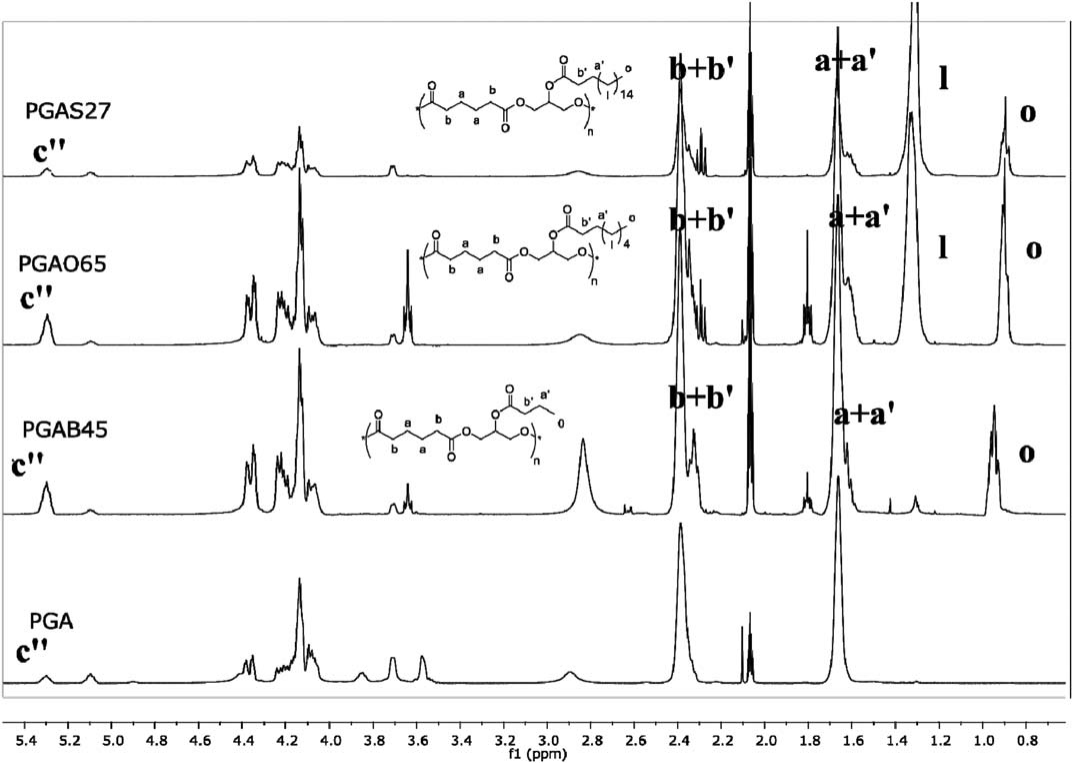
Stacked ^1^H NMR spectra of PGA homopolymer and corresponding acylated derivatives.

^1^H NMR spectra of C_4_, C_8_, and C_18_ PGA polymers showed the appearance of a triplet corresponding to the fatty ester CH_3_ end at 0.9 ppm as well as signals for methylene moieties at 1.3 ppm for C_8_ and C_18_ variants (Fig. 2). PGA functionalization occurs with formation of additional trisubstituted glyceryl repeating units, which are quantified by comparing the integral of its diagnostic peak at 5.30 ppm (peak c″') and those of the PGA backbone. Namely the integral value of c″ increases with the grafting functionalization, resembling a fully esterified glycerol moiety. The acylation degree was calculated employing the equation:

$$\frac{\int C^{''}\left(in\;acylated\;PGA\right)-\int C^{''}(in\;PGA\;starting\;material)}{\int C^{''}\left(in\;acylated\;PGA\right)}\times \;\frac{acyl\;chloride\;fed\;mols}{-OH\;mol\;in\;fed\;PGA}\times 100$$

The above equation can be used when the integral of the methyl protons, on the lateral chain, is set with a full value of 3. On the basis of this assumption, the integral of a + a′ is set assuming a′ equal to 2 protons. By subtracting 2 from the integral of a + a′ a new integral value of a is extracted. This latter value has been used to calculate the relative value of c″ in each modified PGA spectra and time by time in the PGA spectrum (e.g., theoretical target 40%, acylation conversion equal to 85%, therefore acylation acylated amount is 34% mol/mol). Acylation efficiencies – the proportion of acid chlorides successfully conjugated to PGA – ranged from 60% to 92%.

### Thermal Analysis: DSC

**Figure 3:**
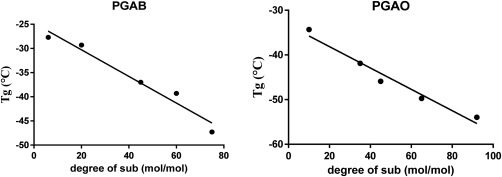
T_g_ trends versus (PGAB) butyric residues amount and (PGAO) octanoic residue amount. In both cases *T*_g_ value decreases with degree of functionalization increment.

The thermal transitions, particularly the glass transition temperature (*T*_g_), can be affected by possible interactions between hydrophilic and hydrophobic segments. All of the PGAs synthesized in this study were characterized by Differential Scanning Calorimetry (DSC) before and after conjugation with C_4_, C_8_, C_18_ alkyl chains (Tables 1 and 2). Thermal properties were investigated to evaluate how grafting affects phase segregation. The modified material sets PGAB and PGAO showed only a glass thermal transition suggesting an amorphous behavior like the PGA backbone. *T*_g_ values decreased with increased acylation conversion in both the PGAB and PGAO series (Fig. 3). This effect might be attributed to a decrease in intermolecular interaction, mainly hydrogen bonds amongst free OH moieties.^26^ In passing from C_4_ to C_8_ grafting, glass transition temperature decreased as the number of carbon atoms increased along the alkyl chains in the side groups. This latter result could be interpreted by considering a major effect of long carbon chains on the polar intermolecular interactions of the main polymer ester backbone.^26^ These two sets of comb‐like polymers show really low *T*_g_ values (Fig. 3), which means at room or body temperature these materials are in an amorphous‐liquid state. Hence, it might be possible to exploit these properties for producing amorphous polymer–drug blends or Nanoemulsion systems to encapsulate both water soluble drugs and lipophilic drugs.

**Table 1:** Summary of Unmodified PGA and the Thermal Behavior of Its B and O Modifications. Table Shows the Presence of a Single *T*_g_ Transition in Unmodified PGA, and the PGAB and PGAO Sets

Polymer	*T*_g_ (°C)
PGA	−33
PGAB11	−28
PGAB27	−29
PGAB45	−37
PGAB60	−39
PGAB75	−47
PGAO12	−34
PGAO35	−42
PGAO45	−46
PGAO65	−50
PGAO92	−54

**Table 2:** PGAS the *T*_g_ Step Values and in Addition the *T*_m1_, Δ*H*_1_ and *T*_m2_, Δ*H*_2_ of the First and Second Peak, Respectively, are Reported

Polymer	*T*_g_ (°C)	*T*_m_ (°C)_1_	Δ*H*_m_ (J/g)_1_	*T*_m_ (°C)_2_	Δ*H*_m_ (J/g)_2_
PGAS14	−27	40	12	65	23
PGAS30	−21	41	19	63	21
PGAS47	−13	41	26	61	10
PGAS65	−11	41	35	62	17
PGAS85	−7	40	42	58	21

**Figure 4:**
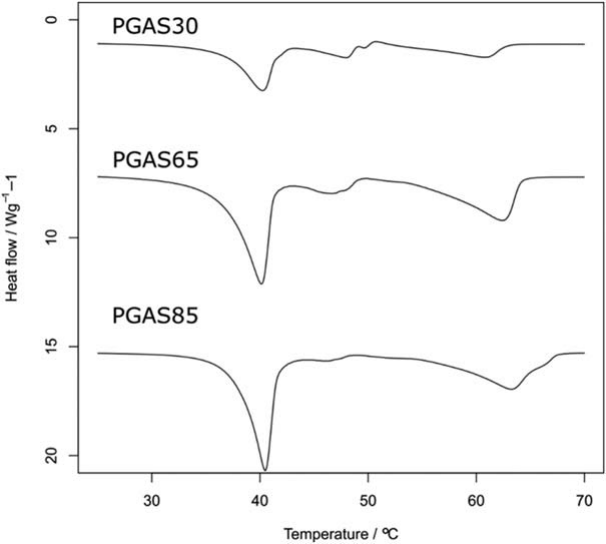
Example of PGAS thermograms. The endothermic transitions area are reported. All the different depicted thermograms show two endothermic transitions. The first transition is around 41 °C and the second one slightly above 60 °C.

**Figure 5:**
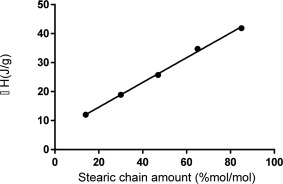
First melting peak area (melting enthalpy) versus degree of stearic functionalization.

At all of the explored side chain conversions PGAS (Supporting Information Figure S1) showed both a *T*_g_ and two endothermic peaks. The presence of both a glass transition and melting transition highlight the presence of two separate phases, namely an amorphous one and a crystalline one. As shown in Supporting Information Figure S1, PGAS85 sample presents the same transitions in every thermal cycle. More than one cycle was repeated on the same sample to evaluate the nature of the endothermic transitions. The presence of these peaks in all the cycles excludes temporary order phenomena or side reactions catalyzed by heating stress. This behavior is characteristic of semi‐crystalline polymers that can be either attributed to melting–recrystallization–remelting mechanisms or to the presence of at least two distinct crystal populations (polymorphs).^19^, ^27^, ^28^ Figure 4 depicts the temperature range between 30 and 70 °C. The first endothermic transition is centered between 40 and 41 °C Table 2 and its area increased with increase in stearic functionalization. A plot of the enthalpies of the first peak versus the substitution degree of the polymers presented here shows a linear dependence with a good correlation indicating a linear increase in crystallinity, *R* ^2^ 0.9978 (Fig. 5). Most likely this first melting peak is strictly related to the amount of stearic side chain packing. This is probably mainly related either to the width (or stiffness) of crystalline lamellae^29^ or to the amount of amorphous phase. Moreover, an increase in the degree of crystallinity with increasing degree of substitution was observed as was also shown elsewhere.^30^ The results demonstrated a variation of Δ*H* with the degree of substitution. Weiss et al.^22^ reported an exhaustive structural and thermal description regarding stearic modifications of PGA at lower molecular weight (PGA_2500_). They have found that PGA_2500_ bearing less than 40% of stearic side chains possess an amorphous state like the unreacted polymer. With an increasing proportion of C_18_ side chains along the main backbone, crystallinity increased linearly with % of acylation. These materials showed only one endothermic peak within the narrow temperature range explored (10–55 °C).

On the contrary, in this work, as shown in Figure 4, the second peak appears much broader, in this case both area and *T*_m_ vary as the amount of stearic side chains change. This latter might be explained by considering different lamellar thicknesses or crystal imperfections commonly found in branched copolyesters.^31^ Taking into account the two extreme functionalization degrees, PGAS14% showed a second peak at 65 °C while PGAS85% presented a *T*_m_ at 58 °C. Since PGA backbone is amorphous, the appearance of endothermic peaks ought to be assigned only to the fatty acid side chains.^22^, ^32^ The presence of two different crystalline phases was also evaluated in AFM measurements. It is probably caused by phase and nanophase segregation between polymer backbone and side chains followed by crystallization of the latter.^20^, ^26^ On increasing the substitution, an increase in the *T*_g_ values was also observed (Table 2). The increase in *T*_g_ related to PGA backbone, due to an higher fatty acid chain interaction, high steric hindrance amongst polymer chains and high rigidity due to alkyl chain cooperativity. Indeed, if phase mixing occurs, the mobility of the segments decreases with a consequent increase in *T*_g_.^33^

### FT‐IR Analysis

**Figure 6:**
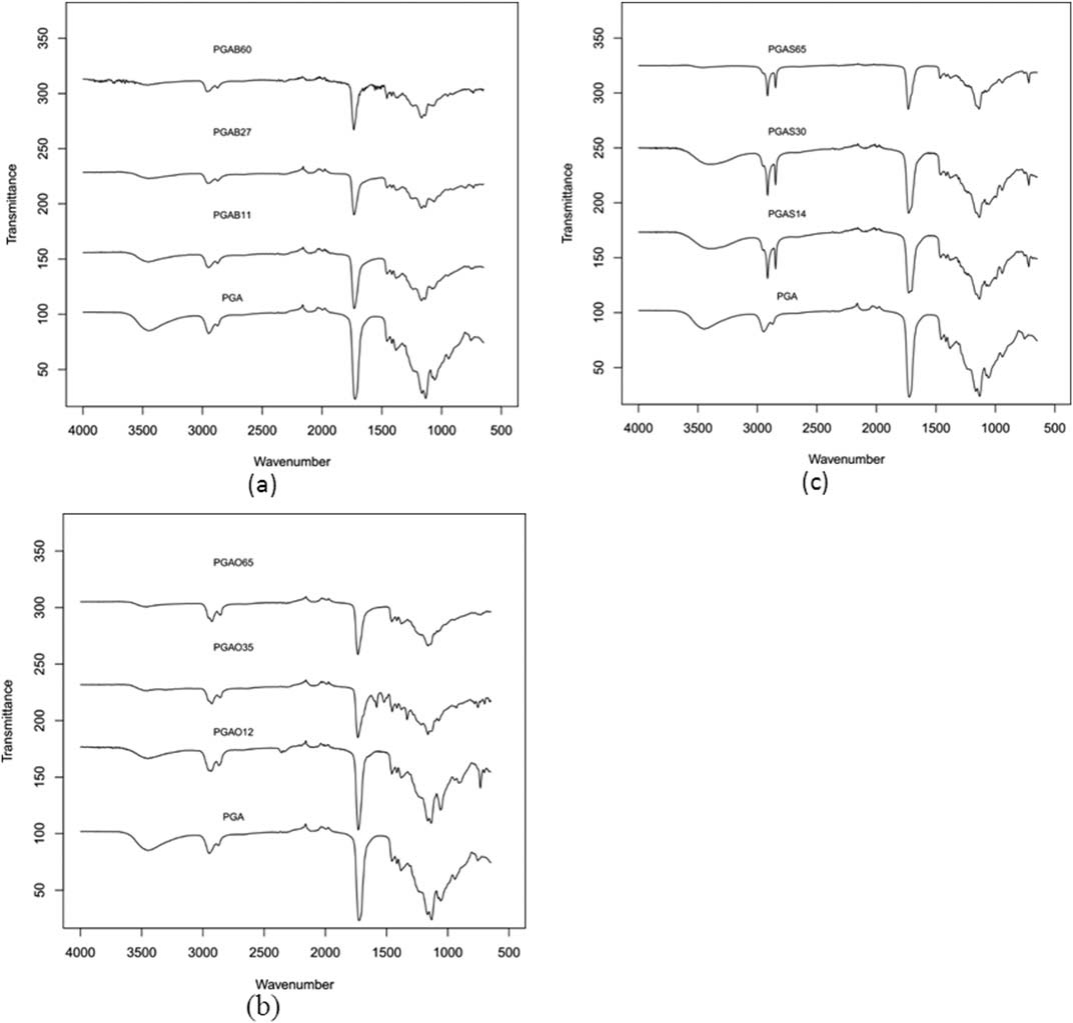
ATR‐IR spectra of PGA compared with (a) PGAB, (b) PGAO, and (c) PGAS series in the spectral range between 4000 and 650 cm^−1^. Transmittance offset has been applied for presentation purpose.

Both PGA polymeric precursor and acylated derivatives were analyzed by FT‐IR (Fig. 6). The spectrum of PGA showed a broad peak for the –OH stretching at 3450 cm^−1^ was likely due to amorphicity of the material and hydrogen bonds,^34^ symmetric and asymmetric stretching of alkyl C—H bonds between 2950 and 2870 cm^−1^, C=O carbonyl group stretching at 1700 while at 1380 cm^−1^ CH bending is present and around 1130–1060 cm^−1^ the C—O—C stretching peak appears. OH bending of free primary and tertiary alcohols appeared at 1075–1055 cm^–^ 1, respectively. Acylation alters particular features of the IR spectrum of PGA and it is possible to qualitatively and semi‐quantitatively associate such changes to % of esterification. In all PGAB, PGAO, and PGAS polymer sets, OH, CH, and C=O stretching showed a net wavenumber shift ranging from 15 to 100 cm^−1^ due to H‐bond rearrangements and different nonpolar interactions amid fatty acid chains. In addition to wavenumber shifts, O—H_υ_ intensity decreased as grafting amount increased (Fig. 6). Hence, FT‐IR spectra also permitted a qualitative assessment of the extent of PGA acylation degree by monitoring the ratio between the intensity of the –OH peak and that of the carbonyl group, which decreased with the increasing degree of functionalization. This observation was true for all sets of different grafted alkyl chain esters synthesized. An example regarding the observed variants is reported in Supporting Information FigureS2. Further to these, a new absorption can be seen at 720 cm^−1^ related to methyl bending (rock vibration) from pendant alkyl chains in addition to PGA signals (Fig. 6).

**Figure 7:**
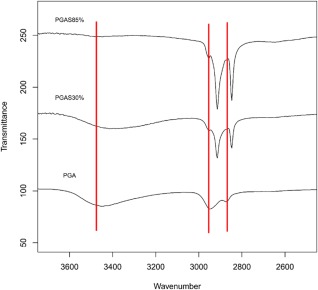
PGA/PGAS O—H and C—H stretching spectral region. The stacked ATR‐IR traces depict both reduction of intensity and shift of the mean wavenumber associated with O—H_υ_ (∼3450 cm^−1^). For C—H_υ_ (∼2900 cm^−1^) a variation of peak shape is shown, likewise a shift in wavenumber. Transmittance offset has been applied for presentation purpose.

**Figure 8:**
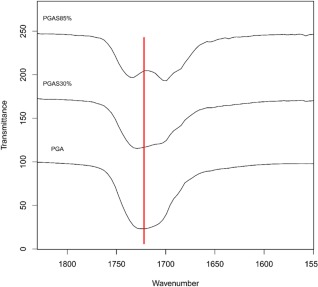
PGA/PGAS C=O stretching spectral region. The stacked ATR‐IR traces depict a shift of the mean wavenumber likewise a variation of peak shape.

In addition to these observations the PGAS series also showed, two strong and sharp stretching absorbances at 2915–2845 cm^−1^ due to CH symmetric and asymmetric stretching of long carbon chains Figure 7. The shape variation of the carbonyl stretching moiety can be observed in Figure 8. Particularly the C=O_υ_ peak becomes sharper, which might be interpreted as qualitative evidence of crystallinity of the PGAS polymer set,^35^ supporting the DSC data for this set of polymers. As acylation conversion increases, the carbonyl stretching peak separates into two peaks (Fig. 8). In addition, the presence of two carbonyl stretching peaks might be traced to two different crystal structures.^35^ To support this evidence, Polarized Microscopy is reported later in the work. Unlike PGAS, both PGAB and PGAO series showed peak shapes similar to unreacted PGA. It is possible to assume that the retention of broad shapes could be a sign of their amorphicity.

### Surface Analysis

#### Contact Angle

**Figure 9:**
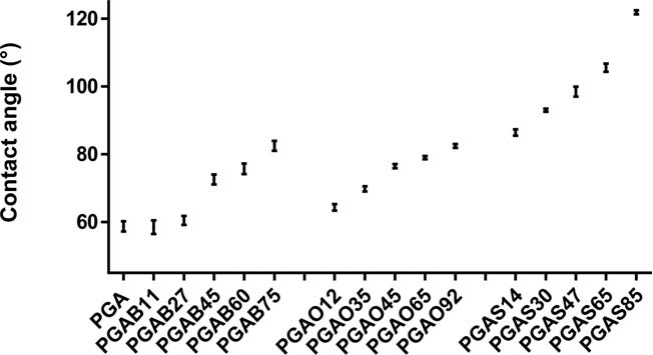
Contact angle trend against degree of functionalization and side chain length. PGA is reported as a reference.

The contact angle made between a water droplet and the surface of a material (WCA) can be used to measure how readily water wets the surface of that material. Increasing the amount of pendant substituents will reduce the number of free hydroxyl groups. In practice it acts as a measure of the hydrophobic or hydrophilic character of that surface on the length scale of the water droplet, with high contact angles corresponding with hydrophobic surfaces, and low angles hydrophilic surfaces. Figure 9 presents the measured WCA of the PGA polymer and its B, O, and S acylated derivatives. The reproducibility of the measurements is good, as indicated by the error bars. PGA possesses an experimental Θ of 58.7. For all C_4_, C_8_, and C_18_ acylated polymers, contact angle Θ mean values increased with the degree of substitution (Fig. 9). For similar degrees of substitution, mean contact angle values increased with the length of acyl substituents (Fig. 6). Although there may be some degree of mixing between these segments, a phase separation is likely to occur due to the different physical and mechanical properties of the phases. The extent of this phenomenon depends on the presence of functional groups or side chains in the polymer structure. In addition, the remaining free hydroxyl groups are less available due to different spatial rearrangements, hence a matrix with pendant substituents improves the hydrophobicity and the phase segregation of the material. It follows from this observation that by decreasing the number of free hydroxyl groups, the hydrophobic portion will become dominant over the hydrophilic portion. PGAS showed lower surface wettability due to both its hydrophobic nature and the increased crystalline order present in its microstructure. Also considering C_4_ and C_8_ sets of amorphous polymers, a significant difference in contact angle amongst the different side chains, was absent resulting in nearly identical trends of hydrophobicity for both sample sets. This is likely due to both the nature of the chemistry underlying the coupling reaction with acyl groups and the low cooperativity amongst C_4_ and C_8_ side chains. Esterification of PGA hydroxyl groups is a random process, without any regioselectivity control, that leads to a random conjugation of the pendant chains throughout the polymer backbone.^22^ This fact is known to influence the microphase separation in comb‐like polymers considerably^36^ and, hence, the surface composition. The WCA can be tuned smoothly over a wide range 60–120 degrees within this family of PGA derivatives. Contact angle changes indicate that the chemical substitutions affect the surface of the polymers, and the smooth change in the value suggests that there is no strong segregation of the acyl substituents between the surface and bulk of the material.

#### AFM Characterization

In addition to contact angle measurements, AFM was employed to observe the surface effects at the nanoscale due to side chain modification.

PGA spread easily on the glass surface, forming layers, spherical formations, crystal‐like fingers, and early spherulites (Supporting Information Figure S3). As Peakforce QNM mode was used, so quantitative observations are possible from the various image channels which map real‐time properties measured from tip–surface interactions. For example, the “DMT” channel maps the Young's modulus measured by the Derjaguin, Muller, and Toporov (DMT) method, so that regions of different stiffness are visible.^15^ At least two regions of different stiffness are visible in the DMT channel of PGA, PGAB 11, 60, and 75, as well as PGAO 65 (Supporting Information Figure S4). Interestingly, PGAS 65 appears to have multiple areas of different stiffness, and crystalline‐like segments by topography.

**Figure 10:**
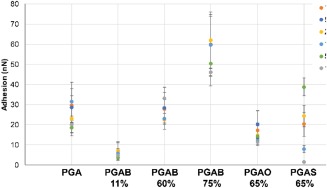
Adhesion values extracted from QNM images of varying image area size. The adhesion is an average of the entire scan area, with the RMS (rq) used as standard deviation.

While WCA probes surface chemistry across the length scale of the water droplet, AFM offers the opportunity of investigating surface topography and chemistry on a variety of much shorter lengths scales. The Peakforce QNM approach is a recent development of probe microscopy which can deliver a variety of quantitative nanomechanical read‐outs. Figure 10 presents adhesion values across a variety of length scales (100 nm–10 µm) extracted from QNM for PGA and for selected PGA derivatives. The range of adhesion values covers approximately one order of magnitude across the samples, with PGAB11% showing the lowest adhesion values (5–10 nN) and PGAB75% the highest (45–75 nN). For individual samples, there appears to be little systematic variation in adhesion as a function of length‐scale probed. The lowest variance in adhesion values as a function of scan area is shown by PGAB11%, with PGAB75%, and PGAS65% showing the largest.

While there are many factors which can in principle affect AFM adhesion experiments,^37^ as our experiments were performed under ambient conditions the primary adhesive force is expected to arise from capillary forces between ambient water adsorbed to the sample surface and the AFM tip.^38^, ^39^, ^40^ Increased adhesion forces are therefore expected for the more hydrophilic samples compared with the hydrophobic ones. In terms of the samples shown in Figure 10 we might therefore expect adhesion forces to vary in essentially the opposite manner as the contact angles shown in

Figure 9, that is PGA > PGAB > PGAO > PGAS, and to decrease as a function of degree of substitution. The variation between samples with different substituents is however not clear, with tip–surface adhesion ranges essentially over‐lapping for PGA, PGAB, PGAO, and PGAS. Within the PGAB series, there is a clear increase in adhesion from PGAB11% through PGAB60% to PGAB75%. This trend is the opposite of that expected based both on measured WCAs, and also on the expected hydrophilicity from the chemical substitutions (OH → O‐alkyl). Based on the data we conclude that factors other than simply hydrophobic/hydrophilic balance are in play. Other potential factors could include surface topography or changes in surface chemistry, both of which must occur on the short QNM length scale but not on the longer WCA length scale.

To further investigate the surface chemistry, stiffness and topography measurements were extracted from the QNM data (Supporting Information Figures S4 and S5), however no clear trend could be identified. The underlying reason for the increased tip–surface adhesion for the PGAB samples as a function of alkyl substitution therefore remains unclear, and further work will be necessary to clarify this issue. Possible explanations might include nm‐scale surface‐segregation of impurities, nm‐scale segregation of hydrophobic and hydrophilic parts of the molecules due to rearrangement of pendant groups at the surface of the sample.

The largest spread in adhesion data across different size scales is observed for PGAS; in this case, the morphological and mechanical data appears distinctive compared with the other samples. (Supporting Information Figure S4 (p–r)). The QNM images show regions on PGAS with higher stiffness and an ordered layered topography, which may be indicative of crystallinity. It appears that there are multiple regions within the PGAS sample with varied surface properties. The adhesion data spread is therefore likely to be due to the multiple regions displaying changed surface properties, observed *via* the QNM height, stiffness, and adhesion channels (Supporting Information Figure S4 (p, q, and r, respectively)). Similar arguments also apply to the other samples showing differences in data spread which somewhat correlate with the data in Supporting Information Figure SI4: The differences in PGAS65 seem to be likely to be due to varying degrees of crystallinity and this would be consistent with spectroscopic and thermal data. As the other samples do not show this high degree of order with respect to topography and stiffness (Supporting Information Figure S4 (left column and centre column, respectively), these samples may be interpreted as primarily amorphous polymers with some small areas of crystallinity observed.

The use of PeakForce QNM for characterizing polymers such as PGA at the nanoscale is relatively underutilized. While we have observed some interesting phenomena here, further work is needed to fully explore the meaning of these differences.

#### Hot Stage Polarizing Microscopy

**Figure 11:**
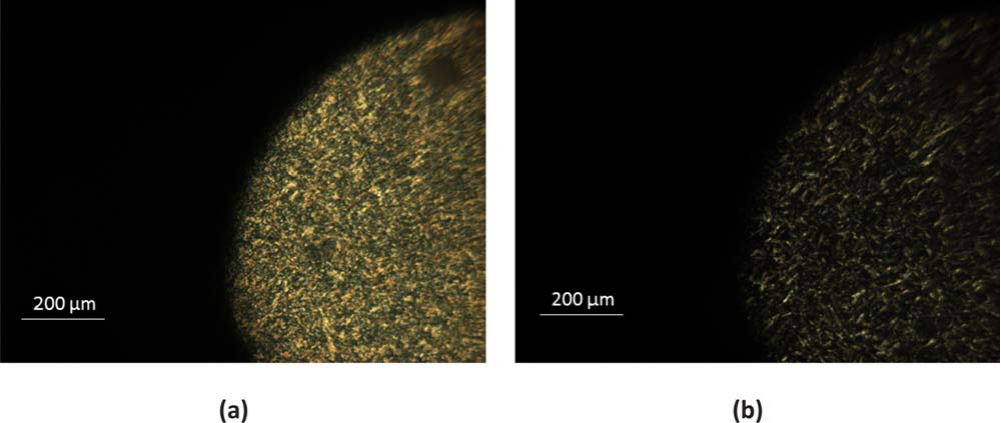
Two polarized pictures of PGAS85 are reported. Figure (a) was depicted at 25 °C, before the first melting peak in DSC, showing an intense birefringence after the first melting. The second photo (b) was taken at 50 °C, despite a reduction in intensity some birefringence is still present.

To clarify the simultaneous presence of different crystal populations, PGAS85 was analyzed by hot‐stage polarized optical microscopy. With the hot stage configuration, it is possible to stress the material to a series of thermal cycles mimicking the DSC experiments with a rate of heating and cooling of 10 °C/min. In combination with the thermal heating/cooling ramps a polarized beam light source was adopted. The light is blocked by inserting a polarizer orientated at 90° to the direction of the illumination. Normally crystalline materials are birefringent and they can strongly interact with a polarized light source, generating evident contrast with the rest of the specimen. During the third heating cycle, pictures were taken at 25 and 55 °C (two isotherms were kept at the two temperatures for 10 min), namely before and after the first melting peak observed in the thermograms (Fig. 11). The picture taken at 25 °C depicts a very bright birefringence. Heating up to 50 °C most of the birefringence is lost yet some optical contrast is detectable, likely indicating the presence of another layer of crystals with a different melting temperature. Over 70 °C, namely, after the second endothermic transition in the DSC no birefringence contrast can be observed.

### NP Formation and Analysis

**Figure 12:**
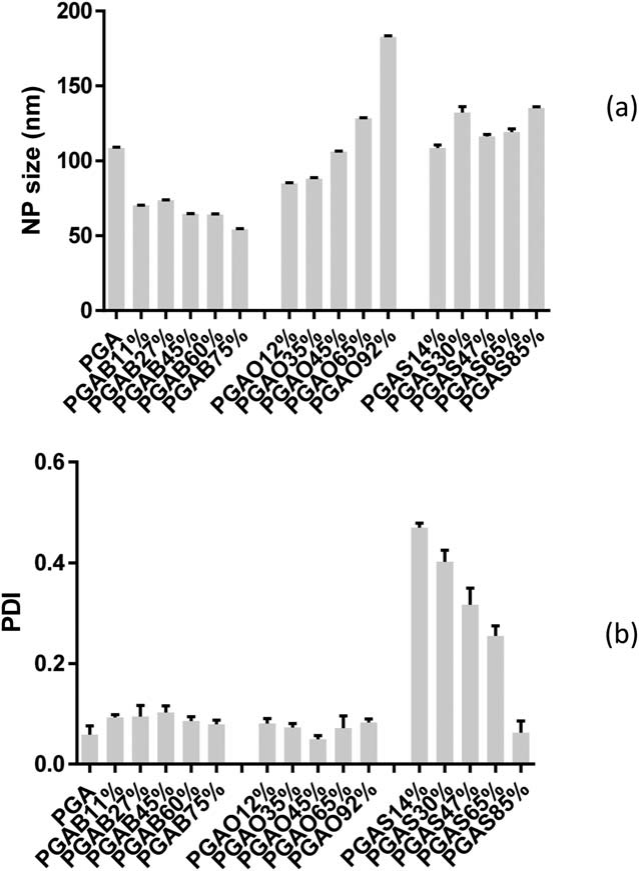
Particle size (a) and polydispersity index (b) of PGA NPs and acylated PGA NPs. Error bars indicate the standard deviation from three measurements. The statistical analysis was performed using IBM^®^ SPSS^®^ Statistics version 21 software.

One purpose of polymer modification is to use the modified polymers as drug delivery carriers. In this study, all synthesized PGA derivatives were employed to prepare NPs for comparison with PGA. The DLS results from intensity plots are graphically summarized in Figure 12. The original data from selected traces are provided in the supplementary information using intensity, volume, and number plots.

At a final polymer concentration of 2 mg/mL, all PGAs could be formed into NPs by self‐assembly without the aid of a stabilizer. The PGA NPs had a particle size of around 110 nm with a very narrow size distribution of less than 0.100 in double distillated water. The modification of PGA with C_4_, C_8_, and C_18_ alkyl chains resulted in different trends in NP properties primarily depending on the chain length of the acyl substituents.

For PGAB even a small substitution resulted in significantly smaller nanoparticles (*p*‐value <0.05) with an increasing degree of C_4_ substitution reducing the size of the PGAB NP even further [Fig. 12(a)]. For PGAO the lower substitution resulted in smaller nanoparticles than PGA, whereas the higher substitutions resulted in larger NP. For PGAS, NP were mostly similar in size to PGA, with the exception of 30% and 85% which were significantly larger (*p*‐value <0.05). For PGAB and PGAO, the polydispersity of the particles was very low, and consistently around 0.1, with some small variations (Supporting Information Figure S6a). The smaller size of PGAB NPs and 12% and 35% PGAO NPs was attributed to the fact that the short acyl chains could easily come in contact with each other and form strong hydrophobic interactions during the particle formation resulting in NP with a compacted core.^41^ However PGAO NPs, at 65% and 92% substitutions, exhibited a very large increase in size, larger size than PGAS NPs This result could be predominantly due to steric hindrance between alkyl chains inside the particle core and thus occupying a larger space in the particle core together with an increased aggregation number.^17^ For PGAS however, there was a very different picture with low stearyl substitutions resulting in a very high polydispersity which became smaller as the amount of pendant groups increased and in which the polydispersity was largely due to additional populations of larger nanoparticles (Supporting Information Figure S6b). In general, we would expect that accommodating the longer pendant chains would increase the particle size, however this may be counteracted by hydrophobic interactions resulting in a more condensed core, and hence overall a similar size to the PGA NP. Compaction would be assisted at higher substitutions by the crystalline nature of the pendant moieties. Variation in size of the PGAS NP populations may be due to an increased aggregation number or aggregation of particles resulting from a number of factors. These may include steric configuration, conformational arrangement and mobility/rigidity of side chains.^42^ It has been reported that the acylation of PGA polymers with C_4_, C_8_, and C_18_ can modulate PGA physical features and its self‐assembling tendency, thus nanoparticle properties. The WCA results indicate that the wettability of the surface of the PGA polymers can be readily tuned, which is likely to be relevant in the use of PGA derivatives in drug delivery. The differences in amorphicity/crystallinity and amphiphilicity balance of modified PGA polymers might affect the particle properties in terms of loading capacity and release behavior of drugs. The PGAB and PGAO polymers showed really low *T*_g_ values meaning that at room or body temperature these materials are in an amorphous viscous‐state. Hence, it might be possible to exploit their features for producing amorphous polymer–drug blends or viscous‐NPs to encapsulate both water soluble drugs and lipophilic drugs. In addition, these new biodegradable materials may be advantageous as potential emulsifier‐free alternatives of solid–lipid nanoparticles (SLNs, solid lipids stabilized with an emulsifier in aqueous dispersion), which have showed promising colloidal drug delivery properties for oral administration of lipophilic drugs.^22^

## CONCLUSIONS

In this work, it has been shown that PGA acylation with C_4_, C_8_, and C_18_ results in comb‐like structures which can modulate polymer properties, physical features and its self‐assembling tendency, thus nanoparticle properties. For all the acylated polymers, regardless of the carbon chain length, contact angle Θ mean values increased with the degree of substitution while considering similar degrees of substitution, contact angle values mainly increased with the length of acyl substituents. AFM was exploited to observe the surface effects due to side chain modification in detail. PGA and its C_4_ and C_8_ modifications spread easily on the glass surface, forming layers and spherical formations. On the other hand, PGAS showed some crystal‐like fingers and early spherulites. PGA, PGAB, and PGAO polymers showed really low *T*_g_ (ranging from −30 to −55 °C) values meaning that at room or body temperature these materials can form nanoemulsions. Interestingly stearic‐grafting modification resulted in semi‐crystalline species where increasing percentages of C_18_ degree of functionalization enhanced crystallinity and *T*_g_ values were much higher due to greater hydrophobic interactions. We then investigated the effects of functionalization on NP formation. Comparing among the different % of substitution of the same substituents, the increasing of degree of C_4_ substitutions reduced the size of the PGAB NPs. Meanwhile, the increasing % of substitution of C_8_ and C1_8_ alkyl chains increased the size of PGAO and PGAS NPs with a greater effect on the octanoyl modified nanoaggregates. The differences in degree of grafting, amorphicity/crystallinity, and amphiphilicity balance of modified PGA polymers might affect the particle properties in terms of loading capacity and release behavior of drugs. Hence, it might be possible to exploit their features for producing amorphous polymer–drug blends or NPs to encapsulate both water soluble drugs and lipophilic drugs. Combining the aforementioned properties with an intrinsic biodegradability and biocompatibility of the main backbone likewise of the side chains, PGA can be considered an excellent alternative to common aliphatic polyesters in the nanomedicine and pharmaceutical fields.

## DATA ACCESS STATEMENT

All raw data created during this research are openly available from the corresponding author (martin.garnett@nottingham.ac.uk) and at the University of Nottingham Research Data Management Repository (https://rdmc.nottingham.ac.uk/) and all analyzed data supporting this study are provided as supplementary information accompanying this paper.

## Supporting information


**Figure S1.** Two thermograms of PGAS85 in the range between −20 and 70 °C. The two main melting transitions are present in both the thermal cycles.
**Figure S2.** Ratio between the intensity of hydroxyl group stretching and the carbonyl group stretching decreases with the degree of functionalization. This particular graph depicts PGAB polymer set trend.
**Figure S3.** AFM height images [with scale bars: *x*, *z*] of (a) PGA [4 μm, 100 nm], (b) PGAB 11% [4 μm, 100 nm], (c) PGAB 60% [1 μm, 100 nm], (d) PGAB 75% [4 μm, 100 nm], (e) PGAO 65% [500 nm, 100 nm], and (f) PGAS 65% [4 μm, 800 nm].
**Figure S4.** AFM images of (a ‐ c) PGA, (d ‐ f) PGAB 11%, (g ‐ i) PGAB 60%, (j ‐ l) PGAB 75%, (m ‐ o) PGAO 65%, and (p ‐ r) PGAS 65%, with left‐most column from height channel, central column from DMT (stiffness) channel, and right‐most column from adhesion channel.
**Figure S5.** DMT (stiffness, a) and height (topography, b) and values extracted from QNM images of varying image area size. Each data point is an average of the entire scan area, with the RMS (rq) used for standard deviation. Samples 1– 6 are PGA, PGAB 11%, PGAB 60%, PGAB 75%, PGAO 65%, and PGAS 65%, respectively.
**Figure S6a** DLS traces of PGA, PGAO12, PGAB11, PGAO92 and PGAB75, PGAS14 and PGAO65. Intensity, volume and number traces.
**Figure S6b.** DLS intensity trace of PGAS14
**Figure S6b.** PGAS47 DLS trace.
**Figure S6b.** DLS Intensity trace of PGAS85.
