# Supplementary material for: Properties of acyl modified poly(glycerol‐adipate) comb‐like polymers and their self‐assembly into nanoparticles
Source: J Polym Sci A Polym Chem. 2016 Jul 8;54(20):3267–78. doi: 10.1002/pola.28215 (PMC5516180; doi:10.1002/pola.28215)
Supplement: Supplementary file 1 — Figure S1. Two thermograms of PGAS85 in the range between −20 and 70 °C. The two main melting transitions are present in both the thermal cycles. Figure S2. Ratio between the intensity of hydroxyl group stretching and the carbonyl group stretching decreases with the degree of functionalization. This particular graph depicts PGAB polymer set trend. Figure S3. AFM height images [with scale bars: x, z] of (a) PGA [4 μm, 100 nm], (b) PGAB 11% [4 μm, 100 nm], (c) PGAB 60% [1 μm, 100 nm], (d) PGAB 75% [4 μm, 100 nm], (e) PGAO 65% [500 nm, 100 nm], and (f) PGAS 65% [4 μm, 800 nm]. Figure S4. AFM images of (a ‐ c) PGA, (d ‐ f) PGAB 11%, (g ‐ i) PGAB 60%, (j ‐ l) PGAB 75%, (m ‐ o) PGAO 65%, and (p ‐ r) PGAS 65%, with left‐most column from height channel, central column from DMT (stiffness) channel, and right‐most column from adhesion channel. Figure S5. DMT (stiffness, a) and height (topography, b) and values extracted from QNM images of varying image area size. Each data point is an average of the entire scan area, with the RMS (rq) used for standard deviation. Samples 1– 6 are PGA, PGAB 11%, PGAB 60%, PGAB 75%, PGAO 65%, and PGAS 65%, respectively. Figure S6a DLS traces of PGA, PGAO12, PGAB11, PGAO92 and PGAB75, PGAS14 and PGAO65. Intensity, volume and number traces. Figure S6b. DLS intensity trace of PGAS14 Figure S6b. PGAS47 DLS trace. Figure S6b. DLS Intensity trace of PGAS85. [file POLA-54-3267-s001.docx]

**Properties of acyl modified poly(glycerol-adipate) comb-like polymers and their self-assembly into nanoparticles**

**Vincenzo Taresco, Jiraphong Suksiriworapong, Rhiannon Creasey, Jonathan C. Burley, Giuseppe Mantovani, Cameron Alexander, Kevin Treacher, Jonathan Booth, Martin C. Garnett**

**
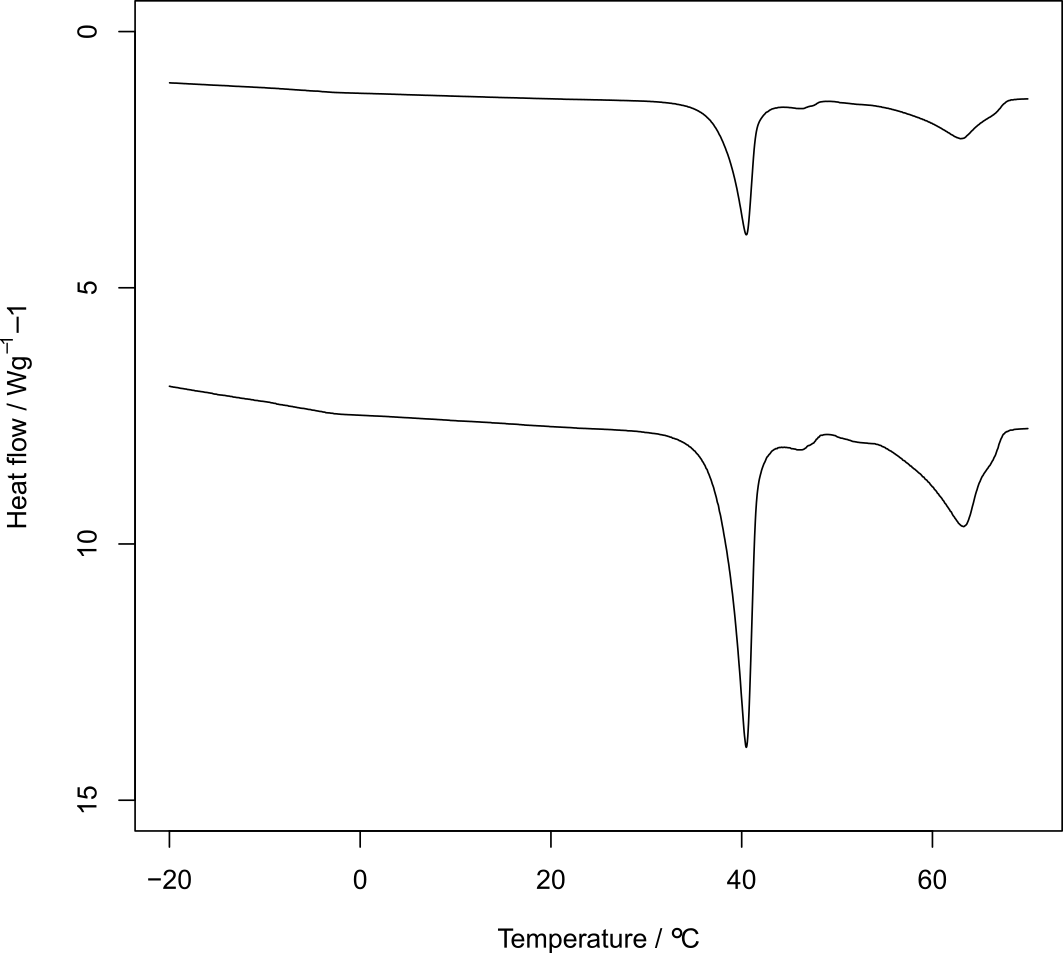
**

**Figure SI1.** Two thermograms of PGAS85 in the range between -20 and 70 ^o^C. The two main melting transitions are present in both the thermal cycles.

**
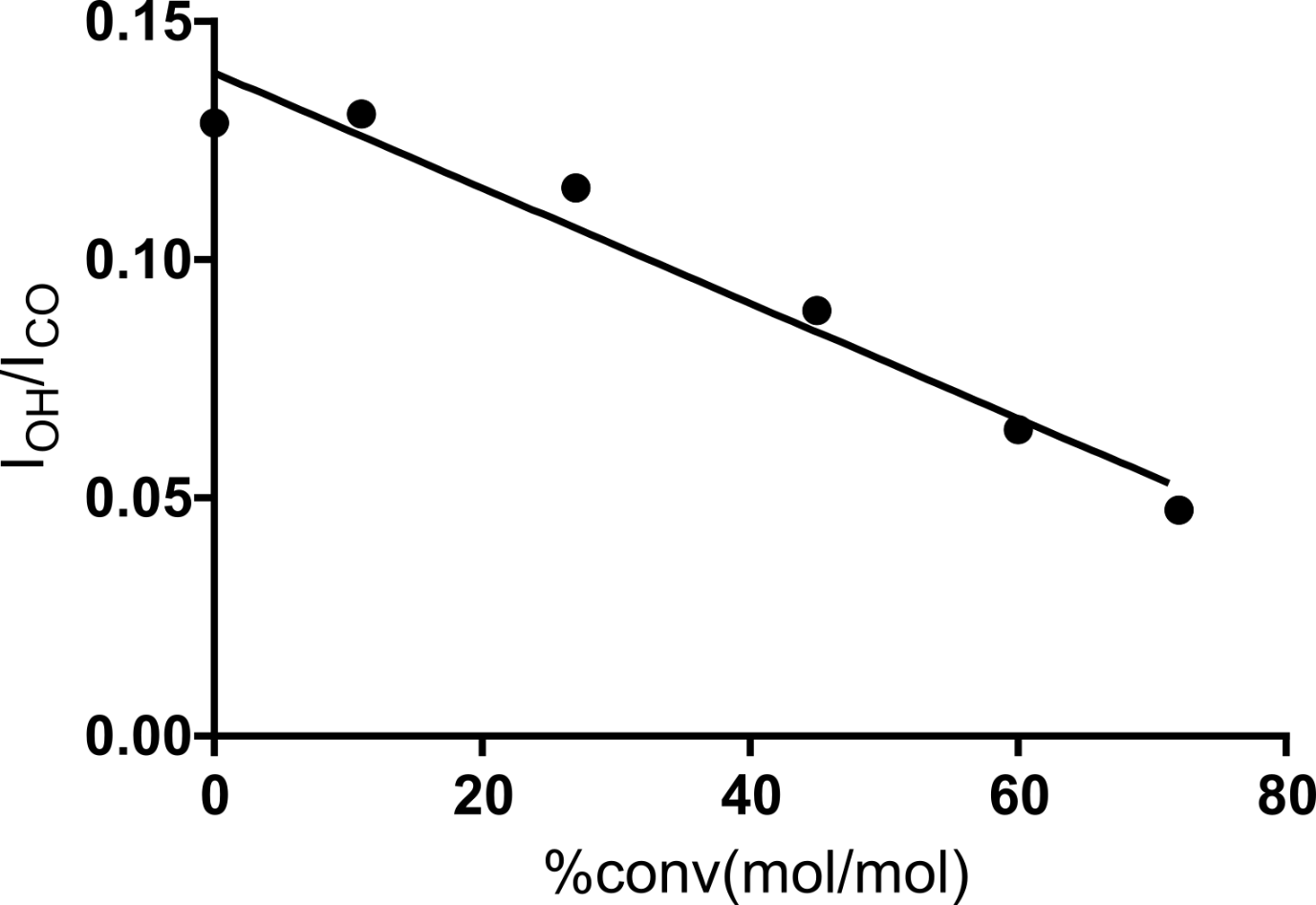
**

**Figure SI2.** Ratio between the intensity of hydroxyl group stretching and the carbonyl group stretching decreases with the degree of functionalization. This particular graph depicts PGAB polymer set trend.


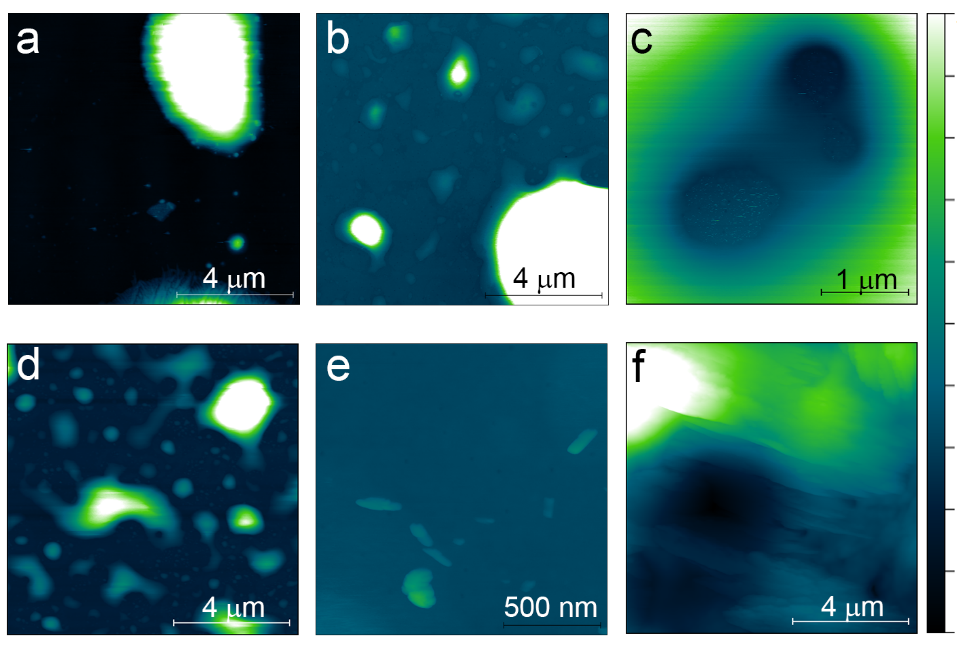


**Figure SI3.** AFM height images [with scale bars: x, z] of (a) PGA [4 μm, 100 nm], (b) PGAB 11% [4 μm, 100 nm], (c) PGAB 60% [1 μm, 100 nm], (d) PGAB 75% [4 μm, 100 nm], (e) PGAO 65% [500 nm, 100 nm], and (f) PGAS 65% [4 μm, 800 nm].


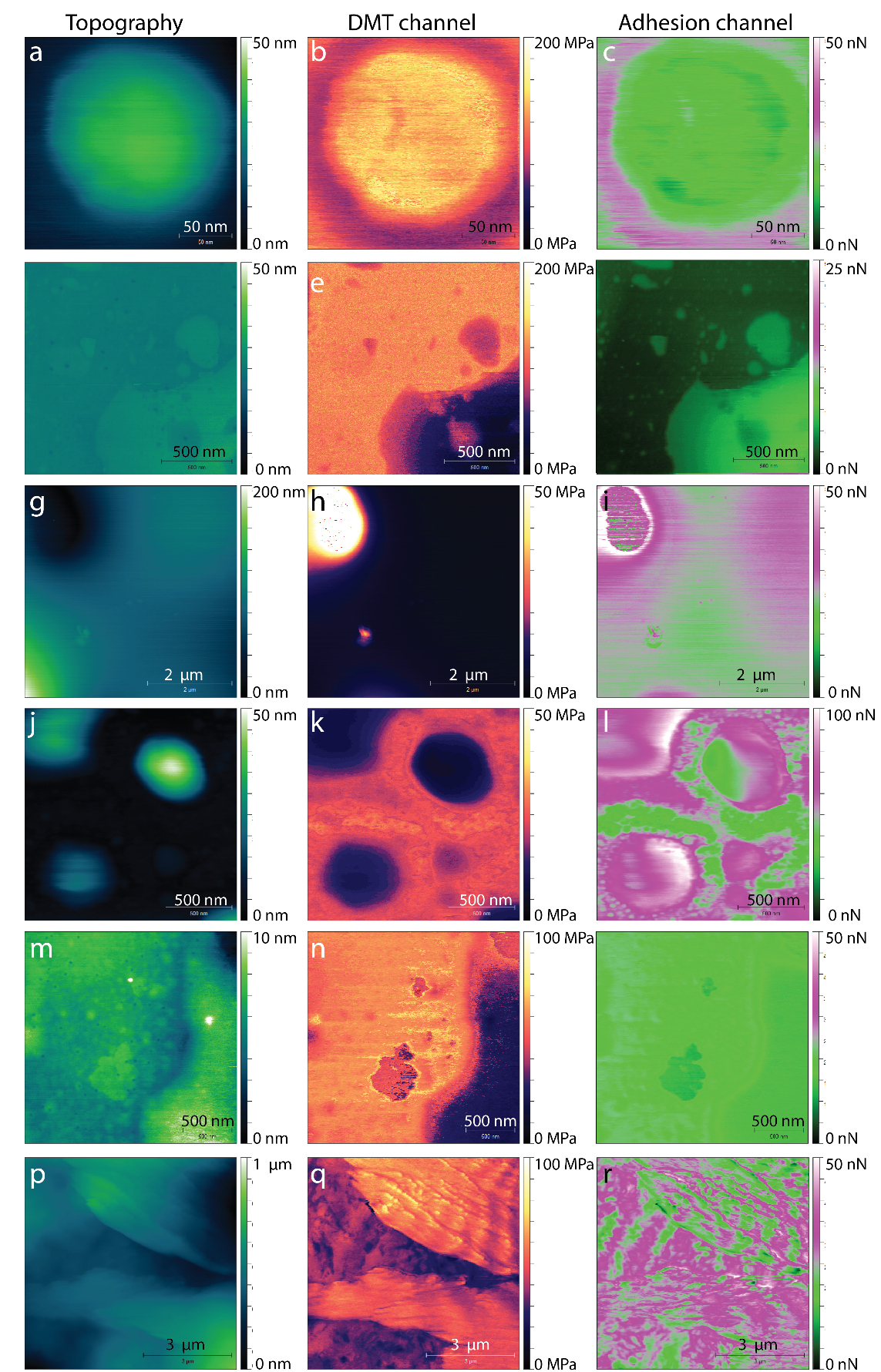


**Figure SI4.** AFM images of (a - c) PGA, (d - f) PGAB 11%, (g - i) PGAB 60%, (j - l) PGAB 75%, (m - o) PGAO 65%, and (p - r) PGAS 65%, with left-most column from height channel, central column from DMT (stiffness) channel, and right-most column from adhesion channel.

b

a

**Figure SI5.** DMT (stiffness, a) and height (topography, b) and values extracted from QNM images of varying image area size. Each data point is an average of the entire scan area, with the RMS (rq) used for standard deviation. Samples 1– 6 are PGA, PGAB 11%, PGAB 60%, PGAB 75%, PGAO 65%, and PGAS 65%, respectively.

**AFM statistical analyses:**

AFM images were analysed using freeware Gwyddion (v2.41, http:/gwyddion.net). For height data, plane subtraction and line correction were carried out, and minimum height was set to zero. For adhesion and DMT data, no data correction was applied. Samples showing a strong topographical artefact were excluded. The ‘statistical quantities’ function was utilised to obtain the average and RMS (rq) values across entire images (512 x 512 pixels) and exported to Excel. Where multiple images of one scale were available, data was averaged and the error was calculated as the square root of the average variance. These data are tabulated as follows where N = number of images used:

|  |  | **10000 nm** | | | **5000 nm** | | | **2000 nm** | | |
| --- | --- | --- | --- | --- | --- | --- | --- | --- | --- | --- |
|  |  | **height (nm)** | **DMT (MPa)** | **Adhesion (nN)** | **height (nm)** | **DMT (MPa)** | **Adhesion (nN)** | **height (nm)** | **DMT (MPa)** | **Adhesion (nN)** |
| PGA | mean | 47.49 | 278.97 | 29.59 | 43.41 | 256.41 | 28.50 | 15.08 | 215.59 | 23.07 |
|  | deviation | 36.1 | 166.9 | 8.3 | 41.4 | 64.0 | 5.9 | 9.4 | 112.0 | 8.4 |
|  | N | 2 | | | 2 | | | 3 | | |
| PGAB 11% | mean | 13.55 | 143.61 | 7.01 |  |  |  | 10.33 | 108.88 | 6.86 |
|  | deviation | 6.8 | 67.7 | 4.7 |  |  |  | 11.1 | 50.4 | 4.4 |
|  | N | 3 | | |  | | | 3 | | |
| PGAB 60% | mean | 273.15 | 7.13 | 27.82 | 84.93 | 13.12 | 28.42 | 44.91 | 29.38 | 22.44 |
|  | deviation | 53.9 | 11.3 | 4.8 | 40.5 | 15.8 | 7.5 | 28.6 | 29.6 | 4.8 |
|  | N | 2 | | | 2 | | | 1 | | |
| PGAB 75% | mean | 28.52 | 19.23 | 59.84 |  |  |  | 21.32 | 20.09 | 62.01 |
|  | deviation | 30.1 | 7.6 | 14.2 |  |  |  | 30.5 | 7.2 | 14.0 |
|  | N | 1 | | |  | | | 3 | | |
| PGAO 65% | mean | 93.85 | 25.79 | 17.19 |  |  |  | 4.62 | 48.47 | 14.84 |
|  | deviation | 41.1 | 21.8 | 3.8 |  |  |  | 2.4 | 15.6 | 2.1 |
|  | N | 2 | | |  | | | 3 | | |
| PGAS 65% | mean | 549.90 | 41.26 | 20.27 | 205.63 | 39.77 | 20.12 | 94.14 | 38.39 | 24.35 |
|  | deviation | 204.4 | 12.2 | 6.0 | 82.3 | 10.0 | 7.0 | 33.2 | 9.0 | 5.3 |
|  | N | 1 | | | 1 | | | 2 | | |
|  |  |  |  |  |  |  |  |  |  |  |
|  |  | **1000 nm** | | | **500 nm** | | | **100 nm** | | |
|  |  | **height (nm)** | **DMT (MPa)** | **Adhesion (nN)** | **height (nm)** | **DMT (MPa)** | **Adhesion (nN)** | **height (nm)** | **DMT (MPa)** | **Adhesion (nN)** |
| PGA | mean | 18.32 | 77.62 | 31.60 | 5.84 | 145.37 | 18.39 | 21.90 | 112.60 | 20.23 |
|  | deviation | 14.2 | 47.6 | 9.4 | 21.0 | 12.9 | 2.4 | 9.7 | 25.8 | 4.2 |
|  | N | 1 | | | 3 | | | 1 | | |
| PGAB 11% | mean | 5.11 | 170.91 | 5.89 | 4.87 | 158.89 | 3.65 | 2.14 | 113.05 | 4.68 |
|  | deviation | 1.2 | 43.3 | 2.3 | 0.5 | 12.4 | 1.0 | 0.9 | 9.9 | 1.2 |
|  | N | 1 | | | 2 | | | 1 | | |
| PGAB 60% | mean | 9.51 | 29.88 | 22.96 |  |  |  | 7.30 | 91.90 | 33.15 |
|  | deviation | 4.1 | 17.1 | 2.7 |  |  |  | 2.3 | 13.4 | 5.4 |
|  | N | 2 | | |  | | | 3 | | |
| PGAB 75% | mean | 11.60 | 18.02 | 59.62 | 39.24 | 13.11 | 50.43 | 10.71 | 7.65 | 46.13 |
|  | deviation | 12.3 | 5.8 | 15.1 | 11.5 | 4.8 | 11.2 | 2.5 | 0.5 | 2.1 |
|  | N | 2 | | | 2 | | | 1 | | |
| PGAO 65% | mean | 9.24 | 74.89 | 12.18 | 7.34 | 91.38 | 14.23 | 5.04 | 36.62 | 11.45 |
|  | deviation | 3.8 | 45.0 | 2.1 | 3.9 | 62.0 | 3.1 | 1.9 | 13.2 | 1.7 |
|  | N | 3 | | | 1 | | | 2 | | |
| PGAS 65% | mean | 46.64 | 44.67 | 7.91 | 7.73 | 24.87 | 38.78 | 11.07 | 30.49 | 1.47 |
|  | deviation | 20.6 | 14.3 | 2.0 | 5.1 | 5.6 | 4.4 | 3.3 | 25.0 | 0.4 |
|  | N | 4 | | | 1 | | | 1 | | |

PGA


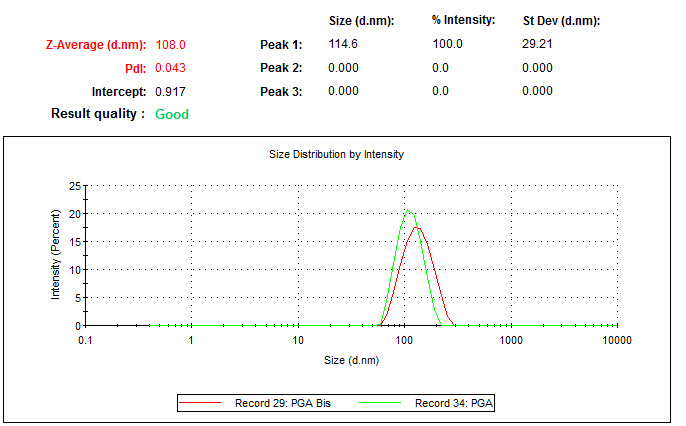


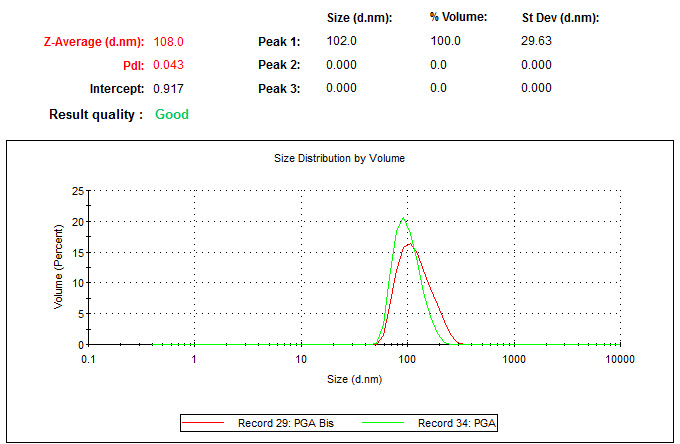


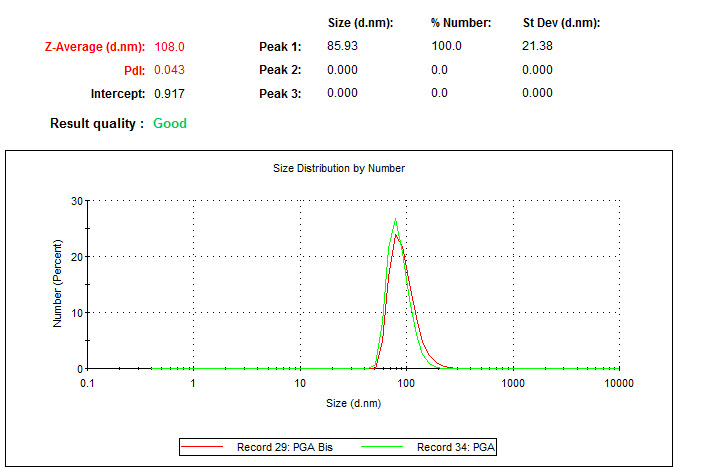


PGAB11


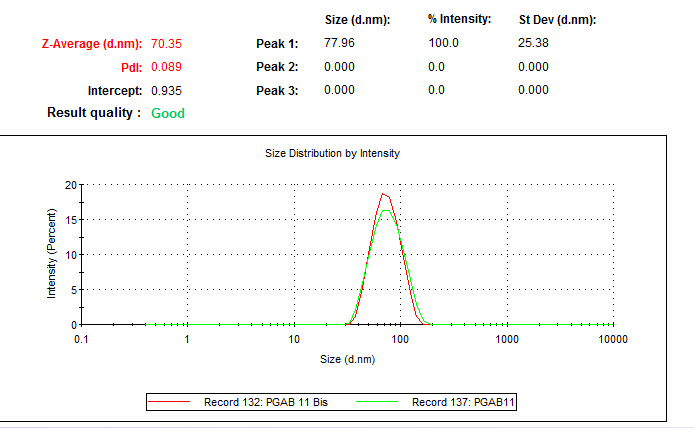


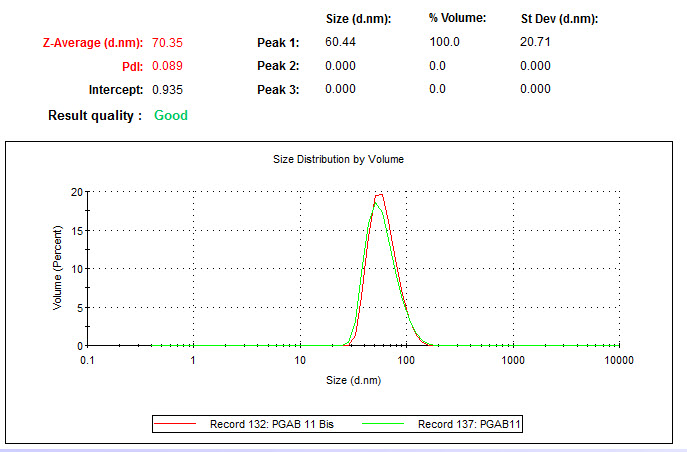


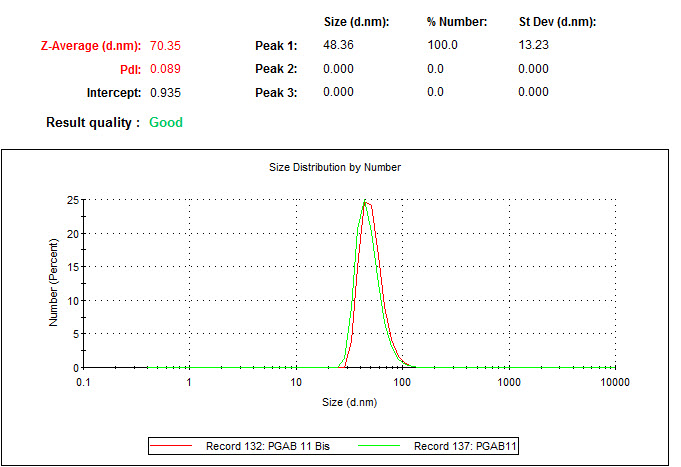


PGAO65


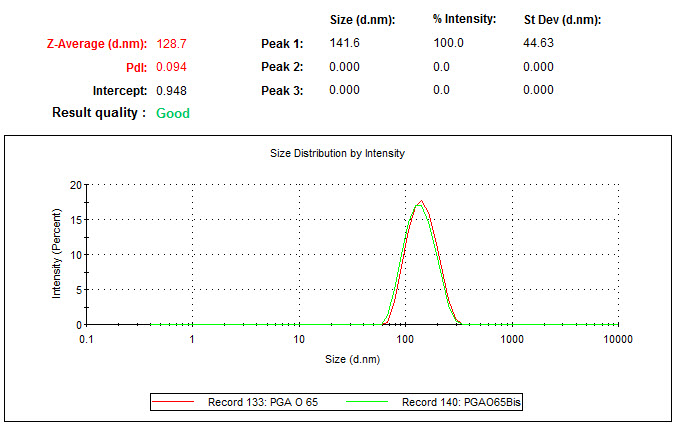


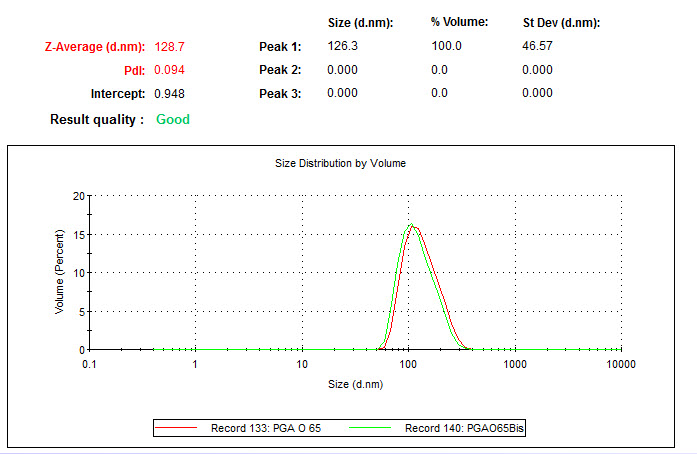


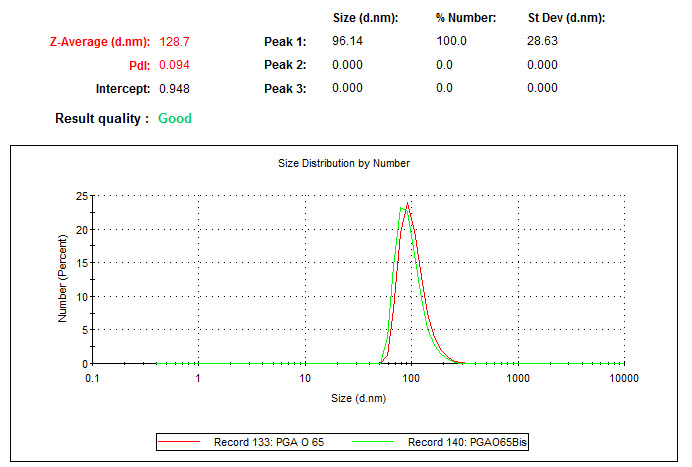


PGAS14


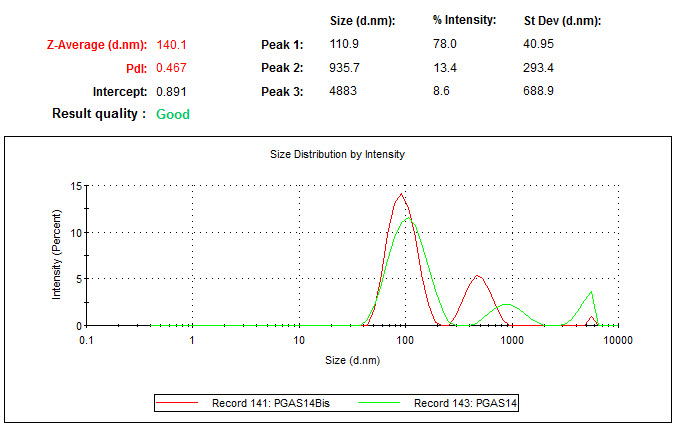


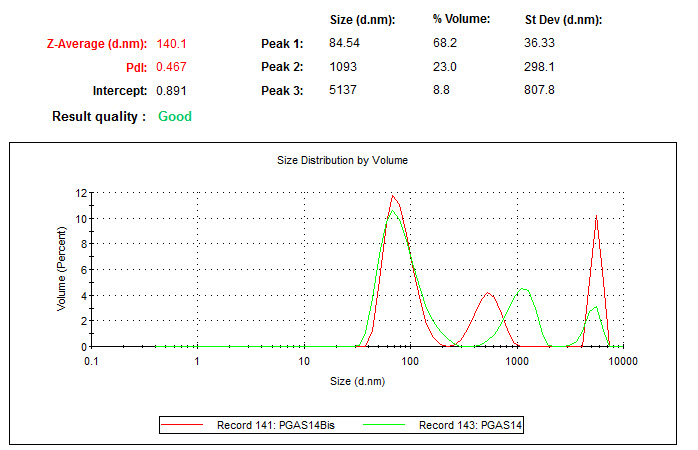


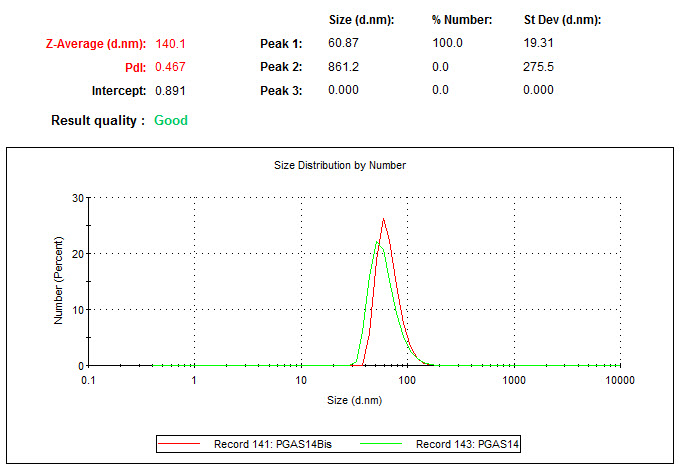


**Figure SI6a** DLS traces of PGA, PGAO12, PGAB11, PGAO92 and PGAB75, PGAS14 and PGAO65. Intensity, volume and number traces.

**Figure SI6b.** DLS intensity trace of PGAS14

**Figure SI6b.** PGAS47 DLS trace.

**Figure SI6b.** DLS Intensity trace of PGAS85.
